# Antimicrobial Drug Resistance in Blood Culture Isolates at a Tertiary Hospital, Uganda

**DOI:** 10.3201/eid2401.171112

**Published:** 2018-01

**Authors:** Henry Kajumbula, Ayako Wendy Fujita, Olive Mbabazi, Christine Najjuka, Charles Izale, Andrew Akampurira, Steven Aisu, Mohammed Lamorde, Richard Walwema, Nathan C. Bahr, David B. Meya, David R. Boulware, Yukari C. Manabe

**Affiliations:** Makerere University College of Health Sciences, Kampala, Uganda (H. Kajumbula, O. Mbabazi, C. Najjuka, A. Akampurira, M. Lamorde, R. Walwema, D.B. Meya, Y.C. Manabe);; Emory University School of Medicine, Atlanta, Georgia, USA (A.W. Fujita);; University of Minnesota, Minneapolis, Minnesota, USA (A.W. Fujita, N.C. Bahr, D.B. Meya, D.R. Boulware);; Mulago Hospital, Kampala (C. Izale); Central Public; Health Laboratories of the Republic of Uganda, Ministry of Health, Kampala (S. Aisu);; University of Kansas, Kansas City, Kansas, USA (N.C. Bahr);; Johns Hopkins University School of Medicine, Baltimore, Maryland, USA (Y.C. Manabe)

**Keywords:** antimicrobial drug resistance, antibacterial drug resistance, antibiotic drug resistance, microbial sensitivity tests, bacteria, Uganda

## Abstract

We summarize antimicrobial drug resistance (AMR) patterns from blood cultures at a tertiary hospital in Uganda. High rates of resistance to first-line antibiotic drugs were observed among *Staphylococcus aureus* and gram-negative organisms. Microbiology services with susceptibility testing should be strengthened to support standardized reporting of AMR data in sub-Saharan Africa.

Antimicrobial drug resistance (AMR) is a global problem (*1**,**2*). However, because of a lack of routine surveillance systems in sub-Saharan Africa, limited data are available describing local AMR patterns (*2**,**3*). In 2015, the World Health Organization adopted an AMR global action plan that called for strengthening knowledge of AMR through surveillance and research (*1*). Understanding local AMR patterns is essential to guide clinical management of infections and to inform health policy. Here we highlight common pathogens and antimicrobial susceptibility patterns from blood culture isolates and their susceptibility patterns at Mulago National Referral Hospital, a tertiary hospital in Kampala, Uganda. 

Blood cultures were collected during June 2013–October 2014 as part of patient care and research studies and processed at 2 microbiology laboratories, Makerere University College of Health Sciences (n = 345) and Mulago Hospital (n = 117). Bacterial identification and susceptibility testing were performed according to 2011 Clinical Laboratory Standards Institute M100 S21 guidelines (https://clsi.org/). Susceptibilities were summarized into a hospital antibiogram using Stata version 12 (https://www.stata.com/stata12/) and Microsoft Excel (Microsoft Corp., Redmond, WA, USA). For organisms with <10 positive isolates, raw numbers were compiled rather than percentages.

In total, 3,197 blood specimens were collected and processed, of which 462 (14%) grew an organism. Gram-positive cocci constituted 60% (279/462) of all isolates. Nearly half (127/279) of these were *Staphylococcus aureus*, of which 32% (41/127) were methicillin resistant, 43% (54/127) were methicillin sensitive, and 25% (32/127) did not have susceptibility data. In our study, 14% of the MRSA isolates (n = 41) were fully susceptible to ciprofloxacin, 55% to clindamycin, 25% to gentamicin, and 4% to trimethoprim/sulfamethoxazole. The remaining susceptibility results are described in the Technical Appendix.

*Enterobacteriaceae* were the most commonly isolated gram-negative organisms, constituting 67% (122/184) of all gram-negative bacilli, including 26% (47/184) *Escherichia coli*, 20% (36/184) *Klebsiella pneumoniae*, 9% (17/183) *Enterobacter* spp., and 3% (5/183) *Citrobacter* spp. We isolated 17 *Salmonella* isolates, 5 *S. enterica* serovar Typhi, and 12 nontyphiodal *Salmonella*. Sensitivity rates of *E. coli* to antimicrobial drugs were as follows: ceftriaxone 33%, ciprofloxacin 39%, chloramphenicol 56%, piperacillin/tazobactam 80%, and imipenem 81%. Sensitivity rates were similar, but lower overall, for *Klebsiella pneumoniae*: ceftriaxone 15%, ciprofloxacin 23%, chloramphenicol 17%, piperacillin-tazobactam 64%, and imipenem 80%. *Pseudomonas aeruginosa* was rarely isolated (n = 3).

We found that a substantial proportion of pathogens isolated from blood demonstrated AMR, principally among methicillin-resistant *S. aureus* and gram-negative organisms. These pathogens were commonly resistant to first-line antibiotic drugs (e.g., fluoroquinolones, penicillins, ceftriaxone) at rates that were much higher than those reported in high-income countries (*4*). 

In Uganda, most of the literature on AMR has focused on single pathogens or subgroups of infections and not on broader, routine surveillance (*5**,**6*). Similar surveillance efforts have been undertaken in other countries in Africa, such as Kenya (*4*). In general, the most common etiologies of bacteremia there, as well as in the United States and several countries in Europe, were similar to those at Mulago Hospital (*4*). The exception was *Salmonella*, which was isolated more frequently in Uganda and Kenya (*4**,**7*).

Although we isolated *Salmonella* spp. in only 17 specimens, we found multidrug resistance (MDR; resistance or nonsusceptibility to >3 different antimicrobial classes) among 3 of 5 *Salmonella* Typhi and 6 of 12 nontyphoidal *Salmonella* isolates. The emergence of MDR *Salmonella* Typhi is well documented in Southeast Asia; however, resistance has been less studied in Africa (*8*). The finding of MDR *Salmonella* Typhi in Uganda aligns with findings from the 2016 Typhoid Fever Surveillance in Africa Program, which found MDR *Salmonella* Typhi in Kenya and Tanzania (*7*).

Limitations of this study include the small sample size of individual species and an urban, tertiary hospital setting with potential selection bias for higher AMR rates. The 3,197 blood cultures collected reflect an average of ≈190 blood cultures/month in an ≈1,800-bed hospital, representing a small fraction of hospitalized patients with infectious diseases. The limited sampling is likely due to patient-incurred costs of blood cultures and limited availability of microbiology supplies. Attempts at laboratory-based AMR surveillance have been attempted in other countries in Africa, including Ghana, Rwanda, and Kenya, with similar challenges (*4**,**9**,**10*). The cost and resource availability in microbiology laboratories are major barriers to large-scale culture testing (*3*), and increased funding for microbiology laboratories is necessary to ensure AMR surveillance. Rapid, non–culture-based technology may be an AMR surveillance option.

In summary, we identified noticeable AMR to commonly available antimicrobial drugs among organisms isolated from bloodstream infections at Mulago Hospital in Uganda. Surveillance using blood culture data can facilitate selecting appropriate therapy and provide hospital antibiograms that can be used for empiric therapy of the very ill. These advantages outweigh the perceived disadvantages of high cost, low yields of recovery, and turnaround time that are frequently considered barriers to performing blood cultures in resource-limited settings. National programs should consider these benefits and prioritize funding for AMR surveillance, and from those data, develop clinical guidelines and national policies to slow the spread of AMR in sub-Saharan Africa.

Technical Appendix Antibiogram of bacterial isolates at Mulago Regional Hospital, Kampala, Uganda, 2013–2014.
